# Developmental origin of the Pair1 descending interneuron

**DOI:** 10.17912/micropub.biology.000707

**Published:** 2022-12-16

**Authors:** Amanda Linskens, Chris Doe, Kristen Lee

**Affiliations:** 1 University of Oregon, Eugene, OR USA; 2 Howard Hughes Medical Institute

## Abstract

Pair1 is part of a *Drosophila* larval locomotor circuit that promotes backward locomotion by inhibiting forward locomotion. We hypothesize that lineage related neurons may function in neuronal circuits together. Testing this hypothesis requires knowing the progenitor of each neuron within this locomotor circuit, and here we focus exclusively on Pair1. During *Drosophila melanogaster* embryogenesis, unique neuroblasts form by inheriting the spatial transcription factors (TFs) expressed in their birth location within the neuroectoderm. We examine the Pair1 neurons using immunofluorescence to determine which neuroblast the Pair1s derive from. Our results show that Pair1 is derived from gnathal neuroblast 5-3 which expresses Gooseberry (Gsb) and Intermediate neuroblasts defective (Ind). When Gsb or Ind were overexpressed in the Pair1 lineage, extra neurons formed with similar Pair1 morphology.

**Figure 1. Expression of Gsb and Ind are important for Pair1 development:**
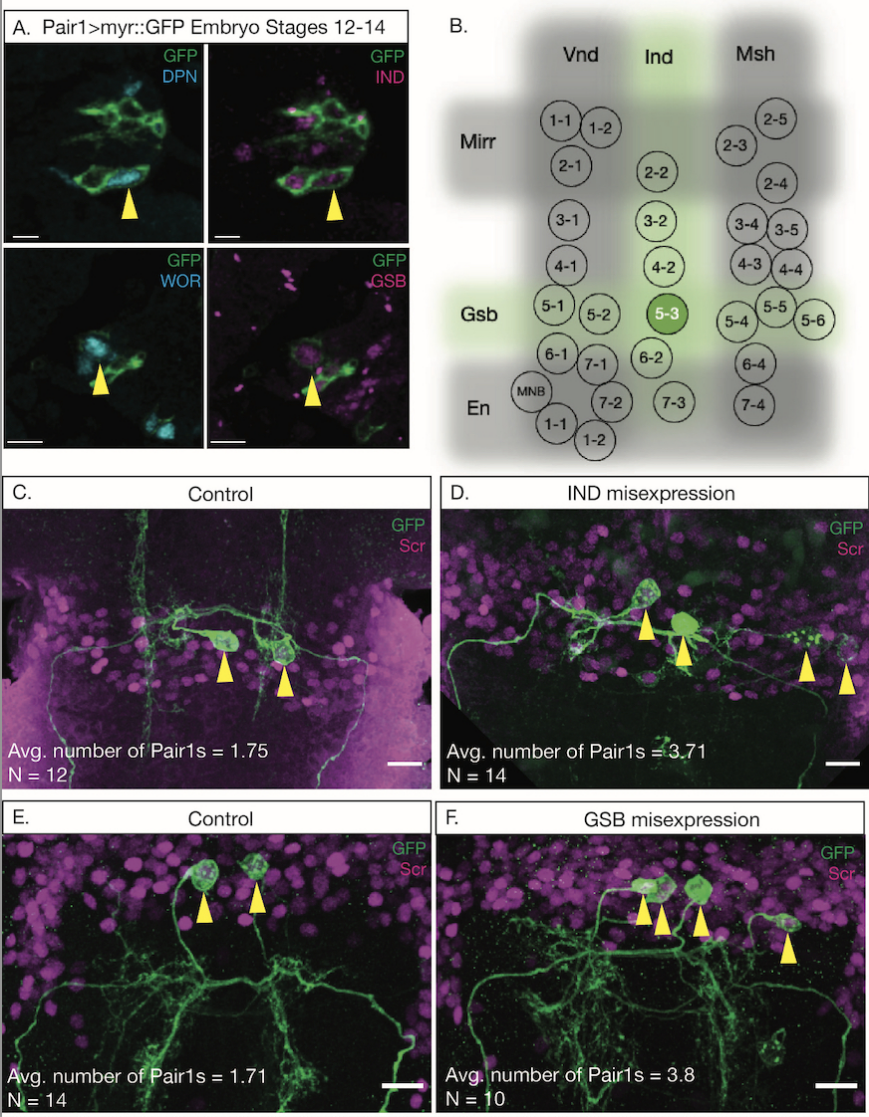
(A) GFP expression via Pair1-Gal4 (Green) colocalizes with neuroblast markers Dpn and Wor (cyan) in stage 12-14 embyros. The Pair1-Gal4 labeled neuroblast (yellow arrow) colocalizes with spatial transcription factors Ind and Gsb (magenta). Single slice, scale bar = 5 mm, n = 12-16. (B) Schematic of *Drosophila* neuroectoderm spatial transcription factors. The only neuroblast positive for both Ind and Gsb is neuroblast 5-3 (green). (C-F) Pair1 cell bodies and morphology (GFP) in control larvae expressing UAS-lacZ (Control; C and E), UAS- *ind* (D) and UAS- *gsb* (F). Cell bodies marked with yellow arrow and the average number of cell bodies reported. 3D projected z-stack, scale bar = 10 mm, n = 10-14.

## Description

**Introduction**

An important question in neuroscience is What mechanisms drive neural circuit assembly? An attractive hypothesis is that neurons with similar developmental origins, e.g. born from the same or similar progenitors, have shared connectivity. *Drosophila* provides an excellent model system for addressing this question, since many neural circuits have been defined and molecular markers for individual neural progenitors (called neuroblasts; NBs) are known. The first step in testing the hypothesis that neurons in a circuit have a common progenitor is to define the neurons in a circuit, and then determine their parental NB. Here we focus on the descending interneuron Pair1, which receives direct excitatory input from the descending interneuron MDN, and provides direct inhibitory input to the premotor interneuron A27h (Carreira-Rosario et al., 2018). This circuit drives backwards locomotion in *Drosophila* larvae: activation of MDN by noxious input results in activation of Pair1, which then activates A27h to pause forward locomotion, which is a prerequisite to initiating backward locomotion (Carreira-Rosario et al., 2018; Tastekin et al., 2018). We would like to know the parental NB for all three neurons, and here we focus on the Pair1 interneuron.

*Drosophila* NBs form a segmentally-repeated and bilaterally symmetrical population, and the spatial factors that are expressed in each NB are well characterized (Crews, 2019), just as the spatial factors that define progenitor populations in the mammalian spinal cord (Jessell, 2000). In *Drosophila* , spatial transcription factors (TFs) create NB diversity. The spatial TFs are first expressed in the neuroectoderm, organized in both rows and columns, which are inherited by the delaminating neuroectodermal cell as it becomes a NB. In the abdominal, thoracic, and gnathal segments, there are seven rows and three columns of NBs, with each named by its row-column number (e.g. NB5-3 is in row five and the third NB from the ventral midline). The combination of spatial TFs gives each NB an unique identity. Spatial TFs expressed in columns include: Ventral Nervous System Defective (Vnd) which occupies the most ventral column, Intermediate Neuroblasts Defective (Ind) which is in a medial column, and Drop (Dr; also called Msh) which occupies the most lateral column (Figure 1B) (Weiss et al., 1998). In addition, there are spatial TFs expressed in rows within the neuroectoderm and NBs, orthogonal to the columnar genes. Spatial TFs expressed in rows include: Mirror (Mirr) which is expressed in rows 1 and 2; Wingless (Wg) in row 5; Gooseberry (Gsb) in rows 5 and 6, and Engrailed (En) which is in rows 6 and 7 (Figure 1B) (Crews, 2019). Taken together, the generation of molecularly unique NBs allows the generation of diverse neuronal populations with NB-specific attributes. Here we identify the parental NB for the Pair1 neuron.

The Pair1 neurons are an ideal model because they are part of a locomotor circuit, their morphology is well-characterized, and there exists a Gal4 transgene that specifically labels the Pair1 neurons (subsequently called Pair1-Gal4). The Pair1 neurons are a set of two bilateral neurons found in the gnathal head segments (i.e. suboesophageal zone) that have axons that project contralaterally into the ventral nerve cord and dendrites that project up into the brain and partially down into the ventral nerve cord (Lee and Doe, 2021). The Pair1 neurons inhibit forward locomotion in both larvae and adult flies (Lee and Doe, 2021). Pair1 expresses the homeodomain TF Bicoid and the zinc-finger TF Hunchback; both are required for Pair1 morphology and behavior (Lee and Doe, 2021; Lee et al., 2022). Although much is known about Pair1 at the cellular and behavioral levels, it is currently unknown which NB the Pair1 neurons derive from, and whether other neurons in the circuit share the same parental NB. Understanding the origins of Pair1 is a prerequisite for asking whether neurons in a circuit have a common parental NB.

**Results**

To determine the Pair1 parental NB, we first investigated whether the Pair1-Gal4 was expressed early enough to label a NB. We found that Pair1-Gal4 colocalized with well-characterized NB markers Deadpan (Dpn; Figure 1Ai) and Worniu (Wor; Figure 1Aii) (Pearson and Doe, 2003). Next, we assessed which spatial TFs colocalize with the Pair1-Gal4 NB. After assessing each spatial TF individually, we determined that the Pair1-Gal4 labeled a NB uniquely expressing the spatial TFs Ind (Figure 1Ai) and Gsb (Figure 1Aii). Taken together, these results show that the Pair1-Gal4 labels NB5-3 (Figure 1B), which is a known gnathal NB (Urbach et al., 2016).

Given that Gal4-driven expression is known to change overtime, we elucidated whether NB5-3 was the parental NB for Pair1 neurons. Overexpression of a spatial TF invariably leads to a duplication of the neuronal progeny expressing that spatial TF (McDonald et al., 1998; Skeath et al., 1995; Weiss et al., 1998; Zhang et al., 1994). We used this concept to determine whether NB5-3 is the parental NB for Pair1 by misexpressing each Ind and Gsb spatial TFs and assaying for ectopic Pair1 neurons. We overexpressed Ind or Gsb individually using the Pair1-Gal4 driver and assessed Pair1 numbers at 76 hours after larval hatching (ALH) when the Gal4 driver is most specifically expressed in the Pair1 neurons (Lee et al., 2022). Control animals overexpressing lacZ (Figure 1C, 1E) have two Pair1 neurons labeled via the Pair1-Gal4 driver, and both these neurons express the Pair1 marker Sex combs reduced (Scr) (Lee and Doe, 2021). When Ind (Figure 1D) or Gsb (Figure 1F) were overexpressed, we observed two additional Pair1-like neurons labeled, for a total of four. All four neurons expressed Scr and had neurites that resembled Pair1 morphology. Taken together, these results demonstrate that NB5-3 is the parental NB for the Pair1 neurons.

**Discussion**

Our data show that the Pair1 interneurons derive from the gnathal NB5-3, which uniquely presents the spatial TF combination of Ind+ Gsb+ and En-. Furthermore, misexpression of Ind or Gsb using Pair1-Gal4 results in duplication of Pair1 neurons, validating these two spatial TFs as being expressed by the Pair1 parental NB. It is somewhat surprising that expression of Ind or Gsb in the Pair1 parental NB results in Pair1 duplication. It is most likely that Pair1-Gal4 expression in some neuroectodermal cells that result in a second NB5-3 forming, but we can't exclude the possibility that Ind and Gsb act as temporal factors, leading to two successive NB divisions generating two Pair1 neurons in each lineage.

We previously showed that Pair1 is born in the Hunchback temporal TF window and that Hunchback functions in mature Pair1 neurons to maintain synapse number, an important component of neuronal identity (Lee et al., 2022). Although the temporal TF cascade is an important factor in determining neuronal diversity, previous studies have shown that spatial TFs act together with temporal TFs to determine neuronal identity. Specifically, in different NBs that create unique progeny, the row spatial TFs (i.e. Gsb) have different open chromatin with Hunchback binding domains (Sen et al., 2019). This suggests that spatial TFs could be more important in determining a neurons identity than previously thought. Understanding how the spatial TFs Ind and Gsb contribute to the temporal TF Hunchback’s role in establishing and maintaining Pair1 neuronal identity could potentially showcase spatial TFs role in establishing, or not establishing, mature neuronal properties. In addition to Ind and Gsb, previous literature has shown that gnathal NB5-3 expresses the genes *seven up* , *unplugged* , and *eyeless* (Urbach et al., 2016). These genes all have well-characterized functions in early development, but their roles outside of development is largely unknown. The Pair1 neurons are an excellent tool to potentially elucidate the role of these genes in the mature nervous system throughout life.

## Methods

*Drosophila* husbandry and stocks

All fly stocks were raised at 24-25°C on a 12-hour light/dark cycle and regularly flipped every two weeks. Flies used for collecting embryos were between the ages of 2 to 9 days after eclosion. Embryos collected and used for experiments were collected in a 25°C incubator for 21 hours until fixed or collected as larvae. Larvae used in the experiments were maintained in a 25°C incubator until dissection. All larvae were dissected after 76 hours after larval hatching. Male and female larvae were not separated. Fly stocks used in this study were obtained from the Bloomington *Drosophila* Stock Center or FlyORF and include GMR75C02-Gal4 (referred to as Pair1-Gal4; BDSC #39886), UAS-myr::GFP (BDSC #32198), UAS-lacZ (BDSC #8529), UAS-gsb (BDSC #42225), UAS-ind (FlyORF F000047)

Embryo Immunostaining

After collecting the embryos, they were coated in bleach and fixed for 25 minutes using 4% PFA. Once fixed the embryos were then cracked using MeOH and allowed to settle. Any floating embryos appearing after this process were discarded. The remaining, properly fixed embryos then were rinsed twice with MeOH and then rinsed once with EtOH. The embryos were then quickly washed using PBS with 0.3% triton and blocked overnight at 4C. After removing the Block, the primary antibodies were applied to the embryos and allowed to stain overnight at 4C. Primary antibodies used in immunostaining include rat anti-Dpn (1:50; Abcam, catalogue number ab195173, Eugene, Oregon, USA), rabbit anti-wor (1:1000; Abcam, catalogue number ab196362, Eugene, Oregon, USA), rat anti-Gsb-d (1:10; gift from the Holmgren lab, Northwestern University, Illinois, USA), rabbit anti-Ind (1:250; described previously(Von Ohlen and Moses, 2009)). The primary antibodies were then removed and stored in a 4C fridge to be used again. The embryos were then either quickly washed using PBS with 0.3% triton three times and washed overnight at 4C or quickly washed two times, washed for at least 30 minutes, and repeated that process once. After removing the wash, the secondary antibodies were applied to the embryos and allowed to stain either overnight at 4C or for at least two hours at room temperature. The embryos were then either quickly washed using PBS with 0.3% triton three times and washed overnight at 4C or quickly washed two times, washed for at least 30 minutes, and repeated that process once. After washing with PBS + 0.3% triton, the embryos were then either mounted with DPX or glycerol. With the DPX mounting process the embryos were washed with HL3.1 and mounted onto slips. The slips were then washed with EtOH series and then washed with xylenes. The slip was then mounted using DPX. With glycerol mounting the embryos were allowed to sit in 50% glycerol at room temperature until all embryos were settled. Once settled the 50% glycerol was removed and 90% glycerol with antifade was added. Embryos sat in 90% glycerol at 4C at least overnight. Embryos were then pipetted onto a slide with a bridge and covered with a coverslip.

Larvae Immunostaining

After dissecting, the larval brain was mounted on a Poly-Lysine slip with HL3.1. This slip was transferred to a well plate and fixed for 12 minutes in 4% PFA. The slip was then quickly washed three times using PBS with 0.3% triton. The brains were then Blocked either overnight at 4C or for at least 40 minutes at room temperature. After removing the Block, the primary antibodies were applied to the slip and allowed to stain overnight at 4C. Primary antibodies used in immunostaining include chicken anti-GFP (1:1500; Abcam, catalogue number ab13970, Eugene, Oregon, USA), mouse anti-Scr (1:10; Developmental studies hybridoma bank, catalogue number 528462, Iowa City, Iowa, USA). The primary antibodies were then removed and stored in a 4C fridge to be used again. The brains were then either quickly washed using PBS with 0.3% triton three times and washed overnight at 4C or quickly washed two times, washed for at least 30 minutes, and repeated that process once. After removing the wash, the secondary antibodies were applied to the slip and allowed to stain overnight at 4C. The brains were then either quickly washed using PBS with 0.3% triton three times and washed overnight at 4C or quickly washed two times, washed for at least 30 minutes, and repeated that process once. After washing with PBS + 0.3% triton, the slips were then washed with EtOH series and then washed with xylenes. The slip was then mounted using DPX.

Imaging and Analysis

All images were taken on the Zeiss 700 or 900 Confocal Microscope using the 63x oil objective lens and were taken as z axis stacks. Images were processed in FIJI using greyscale and composite images. Figure assembly was done with Adobe Illustrator. Images of neuroblasts (Figure 1A) were single slices from the Z-stack. Images for overexpression (Figure 1C-F) were projected into Z-stacks through FIJI using Max intensity.
